# Titanium Carbide (Ti_3_C_2_T*_x_*) MXene as Efficient Electron/Hole Transport Material for Perovskite Solar Cells and Electrode Material for Electrochemical Biosensors/Non-Biosensors Applications

**DOI:** 10.3390/mi14101907

**Published:** 2023-10-06

**Authors:** Theophile Niyitanga, Archana Chaudhary, Khursheed Ahmad, Haekyoung Kim

**Affiliations:** 1 School of Materials Science and Engineering, Yeungnam University, Gyeongsan 38541, Republic of Korea; 2 Department of Chemistry, Medi-Caps University, Indore 453331, Madhya Pradesh, India

**Keywords:** MXene, Ti_3_C_2_T*_x_*, perovskite solar cells, electron transport layers, hole transport layers, electrochemical biosensors

## Abstract

Recently, two-dimensional (2D) MXenes materials have received enormous attention because of their excellent physiochemical properties such as high carrier mobility, metallic electrical conductivity, mechanical properties, transparency, and tunable work function. MXenes play a significant role as additives, charge transfer layers, and conductive electrodes for optoelectronic applications. Particularly, titanium carbide (Ti_3_C_2_T*_x_*) MXene demonstrates excellent optoelectronic features, tunable work function, good electron affinity, and high conductivity. The Ti_3_C_2_T*_x_* has been widely used as electron transport (ETL) or hole transport layers (HTL) in the development of perovskite solar cells (PSCs). Additionally, Ti_3_C_2_T*_x_* has excellent electrochemical properties and has been widely explored as sensing material for the development of electrochemical biosensors. In this review article, we have summarized the recent advances in the development of the PSCs using Ti_3_C_2_T*_x_* MXene as ETL and HTL. We have also compiled the recent progress in the fabrication of biosensors using Ti_3_C_2_T*_x_*-based electrode materials. We believed that the present mini review article would be useful to provide a deep understanding, and comprehensive insight into the research status.

## 1. Introduction

In the present scenario, the design and fabrication of bi-functional or multi-functional materials received extensive attention for various optoelectronic and electrochemical applications [1,2,3]. It is of great significance to summarize the bi-functional properties of such materials towards the development of perovskite solar cells (PSCs) and electrochemical biosensors [4,5,6]. The PSCs have been proven as the most efficient thin film-based photovoltaic technology compared to the conventional solar cells [7,8,9,10]. The poor stability and low charge transport properties of the PSCs are the major concerns for the development of highly stable and high-performance PSCs [11]. Therefore, it has been considered that the stability and charge transport properties of the PSCs can be further enhanced by employing efficient electron/hole transport layers (ETL or HTL) [12,13]. Ding et al. [14] demonstrated the fabrication of tandem solar cells which exhibited a PCE of 21.4%. Chen et al. [15] also reported the enhanced photovoltaic performance of PSCs. Ma et al. [16] optimized the surface morphology and reported the improved photovoltaic performance. Chen et al. [17] reported the passivation and buried interface study of 2D perovskite on ETL and achieved good performance.

The two-dimensional (2D) layered materials such as carbon nitride, hexagonal boron nitride, black phosphorus, transition metal dichalcogenides, and hybrid materials have received enormous attention because of their excellent optoelectronic properties [18,19,20]. The layered materials have been widely explored in various optoelectronic applications such as solar cells, supercapacitors, batteries, light emitting diodes, and photo-detectors [21,22,23,24,25,26,27,28,29,30,31,32,33]. Graphene has been proven as a highly efficient electrode material for various optoelectronic applications [34,35,36,37,38,39,40,41,42,43,44,45]. The 2D layered materials are promising material for their implantation in optoelectronic devices [17]. According to the reported literature, it was observed that the utilization of 2D materials enhanced the charge transport properties and reduced the recombination reactions [43]. The 2D layered materials can offer a perfect uniform surface due to the presence of the inherent confinement in the out-of-plane direction. Thus, 2D layered materials can be suitable as ETL or HTL for the fabrication of high-performance PSCs. Previously, various layered materials such as graphene, MoS_2_, WS_2_, SnS_2_, and TiS_2_, etc., have been reported in the development of PSCs. Tang et al. [45] reported the benign synthetic procedure for the preparation of MoS_2_ and introduced it as ETL for the construction of PSCs. The introduction of MoS_2_ significantly enhanced the photovoltaic performance of the fabricated PSCs and an interesting power conversion efficiency (PCE) of 20.55% was achieved. Other work also demonstrated the use of tungsten disulfide (WS_2_) as ETL and reported a decent PCE of 12.44% [46].

Recently, Yin et al. [47] have proposed an exfoliation method for the preparation of titanium disulfide (TiS_2_) films as ETL for the development of PSCs which demonstrated a PCE of 17.37%. Tin disulfide (SnS_2_) has a CdI_2_-like layered structure (where Sn atoms are sandwiched between two S atoms) and has been explored as ETL by Zhao et al. [48]. The aforementioned points show that layered materials can be used as efficient ETL or HTL materials to improve the PCE of the PSCs.

In 2011, Gogotsi and coworkers have discovered a new class of 2D materials which is known as MXenes [49]. In general, MXenes are a large family of 2D transition metal nitrides, carbonitrides, and carbides with the general chemical formula of M_n+1_X_n_T_x_ (where n = 1, 2 or 3; M = transition metal such as Mo, Ti, Cr, Hf, Zr, Nb, Ta; X = C or N and T_x_ = surface functional group such as –OH, –O, –F, and –Cl) [50]. MXenes possess excellent physiochemical features such as tunable optical, mechanical, biological, and electrical properties, which makes them a suitable candidate for optoelectronic devices [51,52,53]. Among different MXenes, Ti_3_C_2_T*_x_* has attracted material scientists and electrochemists because of its high transparency, excellent electron mobility, thermal stability, and high electrical conductivity [54]. The work function of the Ti_3_C_2_T*_x_* MXene can be tuned from 1.6 to 6.25 eV by employing different synthetic processes and post-treatments [55]. These features of Ti_3_C_2_T*_x_* MXene open new doors for its application as highly efficient ETL/HTL for PSCs application. The Ti_3_C_2_T*_x_* MXene can be prepared at low temperature, which makes it compatible with PSCs manufacturing technology.

In 2009, Kojima et al. proposed the novel PSCs using methyl ammonium lead iodide (MAPbI_3_) as absorber layers [56]. The PCE of the proposed PSCs was less than 4%. Thus, various efforts have been made by various research groups to further improve the performance of the PSCs. The PSCs consist of various components such as an absorber layer, ETL, and HTL [44,45,46]. Titanium dioxide (TiO_2_) has been widely used as ETL for the fabrication of PSCs, but suffers from the presence of trapping states [45]. Many attempts were also made to overcome such issues using novel electron transport materials. The Ti_3_C_2_T*_x_* MXene has excellent charge-carriers mobility and optoelectronic features, and can be utilized as ETL and HTL for the construction of PSCs. In addition, Ti_3_C_2_T*_x_* MXene also possesses excellent electrochemical properties, conductivity, and high surface area [57]. Thus, Ti_3_C_2_T*_x_* MXene has been used in the fabrication of various sensors and biosensors. In case of sensing-related applications, various enzymatic biosensors and non-biosensors have been reported using Ti_3_C_2_T*_x_* MXene-based electrode materials as electro-catalysts [57,58,59,60].

Herein, we have compiled and discussed the recent progress of Ti_3_C_2_T*_x_* MXene for the development of PSCs and electrochemical biosensors/non-biosensors applications. This review article would be beneficial for the scientific community working in the field of PSCs and electro-analytical sensing applications.

## 2. Synthetic Procedures for Ti_3_C_2_T*_x_* MXene

**Figure 1:**
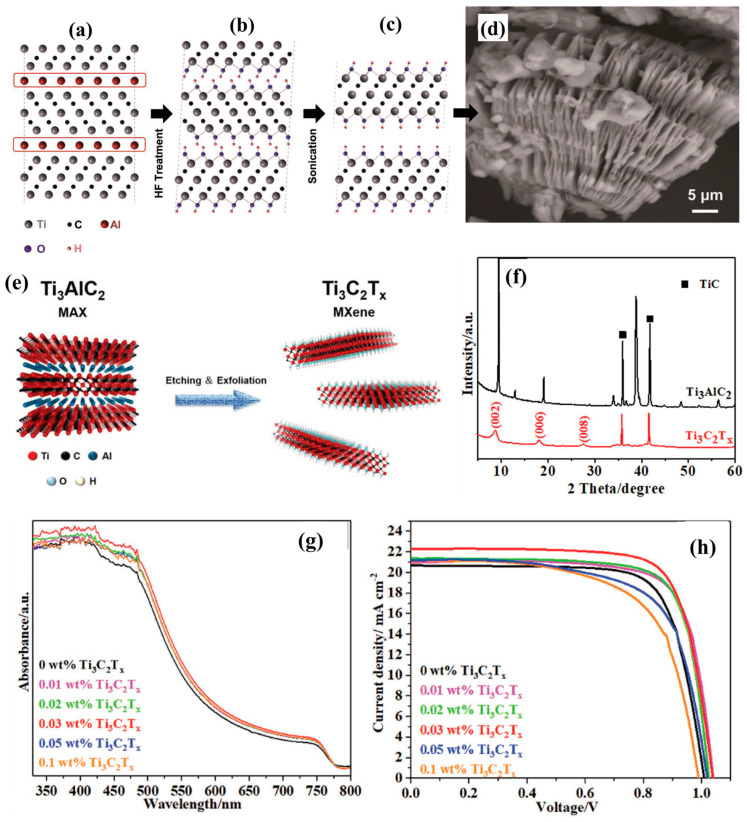
Schematic diagram (**a**–**c**) for the preparation of Ti_3_C_2_T*_x_* MXene and SEM image (**d**) [49]. Schematic illustration for the preparation of Ti_3_C_2_T*_x_* MXene (**e**) [63]. PXRD (**f**) of Ti_3_C_2_T*_x_* MXene. UV-vis spectra (**g**) and J-V curves (**h**) of the Ti_3_C_2_T*_x_*/MAPbI_3_ [77]. Reprinted with permissions from Refs. [49,63,77].

The Ti_3_C_2_T*_x_* MXene was discovered by Gogotsi and coworkers in 2011 [49]. The Ti_3_C_2_T*_x_* MXene was obtained by HF treatment followed by the sonication process, as depicted in Figure 1a–d. The SEM image of the synthesized Ti_3_C_2_T*_x_* MXene has been presented in Figure 1d, which demonstrates the presence of the layered structure of the prepared Ti_3_C_2_T*_x_* MXene. Yu et al. [61] also reported the synthesis of Ti_3_C_2_T*_x_* MXene. In brief, an appropriate amount of LiF was slowly added to the 9 molar HCl using stirring which yielded a homogeneous solution. In further steps, titanium aluminium carbide (Ti_3_AlC_2_) was milled by mortar and pestle and gradually mixed to the above-prepared homogeneous solution. This etching process was continued to 24 h at 25 °C using continuous stirring. Finally, the etched product was washed with DI water using centrifuge. This process was repeated for several times until the pH of the mixture reached above 6. Subsequently, handshaking was used to exfoliate the Ti_3_C_2_T*_x_* into the few-layer flakes. The dispersion of Ti_3_C_2_T*_x_* was degassed with Ar and stored in the fridge at 4 °C [61,62]. In 2019, Yang et al. [63] also reported the preparation of Ti_3_C_2_T*_x_*. In the first step, Ti powder, aluminum powder, and graphite powder were mixed uniformly. This mixture was sintered at high temperature (1650 °C) for 2 h under Ar atmosphere to obtain the Ti_3_AlC_2_ (MAX phase). Then, Ti_3_AlC_2_ powder was grinded and added to the LiF/HCl mixture solution, and kept for the etching process for 24 h. After 24 h, the acidic mixture was centrifuged and washed with DI water until pH of the solution reached 6 and finally Ti_3_C_2_T*_x_* MXene was obtained [63]. The schematic illustration for the preparation of Ti_3_C_2_T*_x_* MXene is presented in Figure 1e. In the past few years, many synthetic procedures have been developed for the preparation of Ti_3_C_2_ MXene. In this section, we have briefly discussed two widely used and efficient etching and exfoliation synthetic procedures as given below.

### 2.1. Etching Method

A previous report showed that the MAX phase of the MXene can be chemically etched by using HF aqueous solution and the etching process can be described as below [64],

2Ti_3_AlC_2_ + 6HF    ⟶    2AlF_3_ + 3H_2_ + 2Ti_3_C_2_ (1)

Ti_3_C_2_ + 2H_2_O    ⟶    Ti_3_C_2_ + Ti_3_C_2_(OH)_2_ + H_2_ (2)

Ti_3_C_2_ + 2HF    ⟶    Ti_3_C_2_F_2_ + H_2_ (3)

The Ti_3_C_2_T*_x_* MXene can be prepared by the etching of Al from the Ti_3_AlC_2_ phase using HF as an etching agent. Studies have shown that H and F radicals broke down after being adsorbed onto Ti atoms, leading to the weakening of Al-Ti bonds. This resulted in the creation of surface terminals and, eventually, the formation of Ti_3_C_2_T*_x_* [64,65,66]. Because chemical etching operates under kinetic control, it is necessary to take into account various significant reaction factors such as time, temperature, and the concentration of HF. It was documented that achieving proper etching of a significant quantity in M_n+1_AX_n_ demands extended etching duration, a relatively elevated etching temperature, and a low pH level. The primary resulting compound during the etching process is AlF_3_ (as shown in Equation (1)), which is insoluble in water. Hence, the careful selection of suitable temperature and duration is crucial to prevent the formation of AlF_3_ precipitates. Moreover, the direct use of HF is very dangerous. Thus, to avoid the direct utilization of toxic HF, Halim and colleagues opted for a safer and less intense approach by using NH_4_HF_2_ instead of the hazardous HF for their etching process [67]. Similarly, Ghidiu et al. [68] took an alternative route by introducing Ti_3_AlC_2_ powder into a mixture of LiF and HCl (6 M) to produce Ti_3_C_2_T*_x_*. They conducted the reaction at 40 °C for 45 h, and their findings highlighted that the presence of both fluorine ions and protons played a crucial role in the successful etching. Expanding on this method, Lipatov et al. [69] adjusted the ratios of MAX and LiF (MAX: LiF = 1:5 or 1:7.5), revealing that an excess of LiF facilitated the etching of Al, along with the insertion of Li^+^. As a result, Ti_3_C_2_T*_x_* MXene nanosheets were achieved, boasting larger dimensions, uniform thickness, and fewer imperfections. The etching procedure essentially converted the compact MAX structure into a loosely arranged, accordion-like configuration, recognized as multilayer MXenes (ML-MXenes). Consequently, a separate exfoliation step became essential to isolate the ML-MXenes into individual monolayers. It is important to mention that wet-chemical techniques are unsuitable for etching nitride-based MAXs because they have a higher energy barrier for formation. Urbankowski and colleagues [70] came up with an alternative approach involving molten salt (like potassium fluoride, lithium fluoride, and sodium fluoride) to create Ti_4_N_3_-based MXene. They achieved this by selectively removing aluminum from Ti_4_AlN_3_ at 550 °C in an argon environment. The leftover fluoride in the produced powder was subsequently eliminated using a solution of sulfuric acid (H_2_SO_4_).

### 2.2. Exfoliation Method

In 2013, the process of exfoliating MXene nanosheets into individual layers involved the introduction of large organic molecules into the spaces between the layers of the accordion-shaped structure [71]. This was followed by either mechanical vibration or the use of ultrasonic energy [72]. Common substances used to create these spaces, known as intercalants, included tetrabutylammonium hydroxide (TBAOH), dimethyl sulfoxide, hydrazine, urea, and NH_4_^+^ [73,74,75,76]. For instance, researchers led by Chia employed TBAOH as the intercalant to exfoliate Ti_3_C_2_T*_x_* MXene from products obtained through HF etching [59]. The process involved expanding the MAX powder containing larger flakes after removing Al. This resulted in Ti_3_C_2_-HF, which was then separated into individual or a small number of MXene layers by reducing the interaction between layers using TBAOH. Alternatively, when NH_4_HF_2_ was used as the etchant, the accordion-like multilayer Ti_3_C_2_T*_x_* MXene already contained cations like NH_4_^+^ within the aqueous solution.

### 2.3. Structural and Physiochemical Properties of Ti_3_C_2_T*_x_* MXene

The Ti_3_C_2_T*_x_* MXene consists of an interlayer region, intra-layer skeleton region, and surface terminating groups. The Ti and C atoms are stacked in the intra-molecular skeleton region to form the ionic bonds. In case of the interlayer region, the interaction between the layers was connected through the hydrogen bonding between either F/O atoms or van der Waals forces between O and F atoms. It was also observed that the strength of the hydrogen bonding depends not only on the number or distribution of –OH groups but also on the orientation of –OH. A large number of termination groups are also distributed on the Ti_3_C_2_T*_x_* MXene surface. The properties of the Ti_3_C_2_T*_x_* MXene can be influenced by the presence of functional groups. The Ti_3_C_2_T*_x_* MXene has good charge carrier mobility, metallic conductivity, and work function, which suggest its potential applications in photovoltaic devices. The transformation of the MAX phase to MXene phase has been illustrated in Figure 1a–c, whereas the scanning electron microscopic (SEM) picture of the MXene is presented in Figure 1d.

### 2.4. Charge Transport Properties of Ti_3_C_2_T*_x_* MXene

The Ti_3_C_2_T*_x_* MXene possesses excellent charge transport properties, which makes it suitable ETL and/or HTL materials for the fabrication of high-performance PSCs. Here are several essential facets related to its properties of charge transport. The Ti_3_C_2_T*_x_* MXene demonstrates excellent electrical conductivity, which is desirable and the most crucial feature for their applications in electronic devices. The presence of the metallic nature in Ti_3_C_2_T*_x_* MXene arises from its unique layered structure, which includes transition metal carbide layers that facilitate the effective charge transport [49].

In addition, delocalized electronic transition states in the Ti_3_C_2_T*_x_* MXene structure contribute to its presence of metallic properties. The presence of metallic Ti-C bonds in the Ti_3_C_2_T*_x_* MXene allows the movement of electrons and is responsible for the high conductivity of Ti_3_C_2_T*_x_* MXene. The charge carriers, such as electrons, experience the high mobility in the Ti_3_C_2_T*_x_* MXene and contribute to its excellent charge transport properties [77]. Thus, it is clear that high carrier mobility is crucial for electronic applications, as it can allow for efficient and fast movements of charge carriers. Due to the favorable charge transport properties, Ti_3_C_2_T*_x_* MXene can be used as efficient ETL or HTL for the development of PSCs [63].

It can be noted that research in the field of Ti_3_C_2_T*_x_* MXene is ongoing, and new findings may further uncover additional details regarding the charge transport properties.

## 3. Ti_3_C_2_T*_x_* MXene in PSCs

### 3.1. Ti_3_C_2_T*_x_* MXene as Additive for PSCs

The Ti_3_C_2_T*_x_* MXene has excellent stability under ambient conditions and moisture. Perovskite materials are sensitive to the moisture and ambient conditions. Therefore, the use of Ti_3_C_2_T*_x_* MXene as additive to the perovskite material may significantly improve the stability of the perovskite materials. Hence, Ti_3_C_2_T*_x_* MXene can be used as additive for the development of highly stable PSCs. Therefore, Ma and his research team investigated the role of the synthesized Ti_3_C_2_T*_x_* MXene as an additive to control the crystallization of the absorber layer (MAPbI_3_; where M = CH_3_NH_3_^+^) for PSCs [77]. Authors used the acidic etching method for the preparation of Ti_3_C_2_T*_x_* MXene. The powder X-ray diffraction pattern (PXRD) of the Ti_3_C_2_T*_x_* MXene showed the well-defined diffraction peaks (Figure 1f) which confirmed the formation of the Ti_3_C_2_T*_x_* MXene phase. Further, authors developed PSCs using tin oxide (SnO_2_) as an electron transport layer and introduced Ti_3_C_2_T*_x_* MXene as an additive to improve the charge transportation process. Authors introduced different weight percentages of Ti_3_C_2_T*_x_* to the SnO_2_ layer and observed that the introduction of Ti_3_C_2_T*_x_* enhanced the optical properties of the SnO_2_ layer (Figure 1g). The developed PSCs with Ti_3_C_2_T*_x_* as an additive exhibited the highest PCE of 16.80% (Figure 1h). This enhanced PCE may be due to the better electron transfer and lower charge transfer resistance.

In another report published in 2019, Agresti et al. [78] proposed novel strategies to tune the work function of the Ti_3_C_2_T*_x_* MXene. The PCE of Ti_3_C_2_T*_x_* MXene incorporated MAPbI_3_-based PSCs, which can be further improved. In this work, authors investigated the critical role of Ti_3_C_2_T*_x_* MXene for work function tuning and interfacial engineering of PSCs. The Ti_3_C_2_T*_x_* MXene with different termination groups (T*x*) was employed to engineer the perovskite/electron transport layer interface and tune the work function of the perovskite light absorber and TiO_2_ electron transport layer. Authors found that MXenes can efficiently tune the work function of perovskite light absorber and electron/hole transport layers without affecting other properties of the perovskite or electron transport layers. This showed the potential applications of MXene in the development of PSCs. However, the critical role of MXenes in the work function or interfacial engineering needs to be further investigated for various optoelectronic applications [79]. In other work, Zhang et al. [80] decorated MAPbBr_3_ by few-layered Ti_3_C_2_T*_x_* sheets by employing the in situ solution growth method.

### 3.2. Ti_3_C_2_T*_x_* MXene as ETL for PSCs

**Figure 2:**
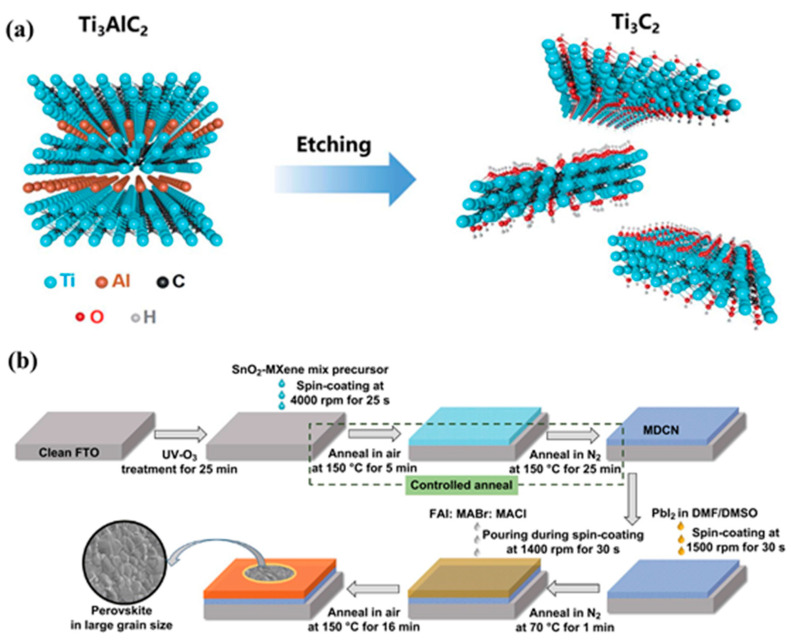
Schematic presentation for the preparation of MXene (**a**) and fabrication of PSCs (**b**). Reprinted with permission from Refs. [82,83].

**Figure 3:**
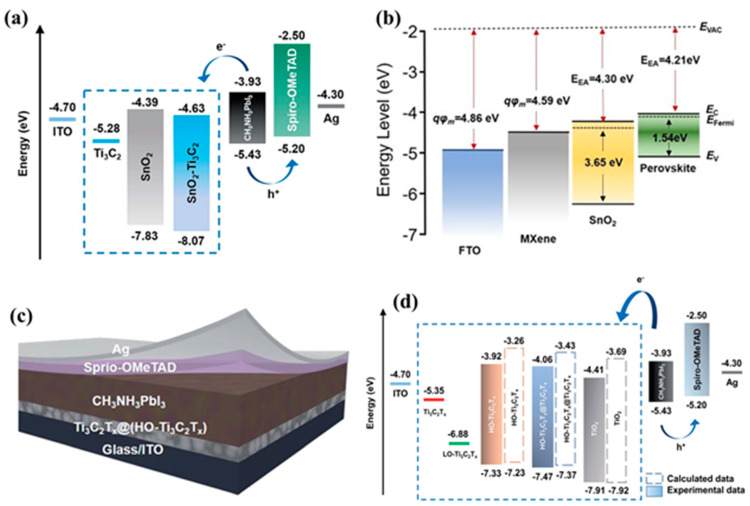
Energy level diagrams (**a**,**b**,**d**) and schematic diagram (**c**) of the MXene-based PSCs. Reprinted with permissions from Refs. [82,84,85].

Since efficient electron transportation is crucial for achieving high efficiency of the PSCs, ETL plays a vital role in the development of high-performance PSCs. The Ti_3_C_2_T*_x_* MXene has excellent properties such as high electrical conductivity, which is a desirable feature for an ETL. The ETL with high electrical conductivity provides the better electron transportation and reduces the electron recombination process. Ti_3_C_2_T*_x_* MXene has suitable electron affinity, which may be useful to collect and transport the electrons efficiently. The physiochemical properties of the Ti_3_C_2_T*_x_* MXene can be tuned or modified through surface functionalization or by incorporating it into composite materials. These features suggested that Ti_3_C_2_T*_x_* MXene may be used as ETL in the development of PSCs. The performance of the PSCs largely depends on the electron transport layer, perovskite light absorber layer, and hole transport layer. Hence, Chen et al. [81] developed Ti_3_C_2_T*_x_* quantum dots to engineer the perovskite/electron transport layer interface and an improved PCE of 21.64% was reported. Yang et al. [82] prepared a novel electron transport layer (Ti_3_C_2_T*_x_*/SnO_2_) to improve the photovoltaic performance of the PSCs. Ti_3_C_2_ MXene was prepared by etching of Ti_3_AlC_2_ as shown in Figure 2a. The developed PSCs using Ti_3_C_2_T*_x_*/SnO_2_ as an electron transport layer exhibited the enhanced PCE of 18.34%. The lowest PCE of 5.28% was achieved for the Ti_3_C_2_ MXene-based PSCs device, while a relatively high PCE of 17.23% was achieved for the SnO_2_-based PSCs device. This showed that the presence of Ti_3_C_2_T*_x_* MXene enhanced the electron transportation and an improved PCE of 18.34% was achieved [82]. The energy level diagram of the Ti_3_C_2_T*_x_*/SnO_2_-based PSCs is presented in Figure 3a. The incorporation of Ti_3_C_2_T*_x_*/SnO_2_ as an electron transport layer not only improves the charge extraction but also enhances the photovoltaic performance of the developed PSCs. Huang et al. [83] also developed a multi-dimensional conductive network (MDCN) electron transport layer using Ti_3_C_2_T*_x_* MXene for the construction of high-performance PSCs (Figure 2b). The enhanced PCE of 18.44% was reported for the MDCN-based PSCs device. Moreover, the developed PSCs device showed good stability in air for more than 45 days. Wang et al. [84] also developed a PSCs device using a Ti_3_C_2_T*_x_* MXene-modulated electrode/SnO_2_ interface. The Ti_3_C_2_T*_x_* MXene was prepared by the chemical exfoliation method and employed as charge transport material. The energy level values of the Ti_3_C_2_T*_x_* MXene were found to be well suited with the energy level values of the SnO_2_ (Figure 3b).

**Figure 4:**
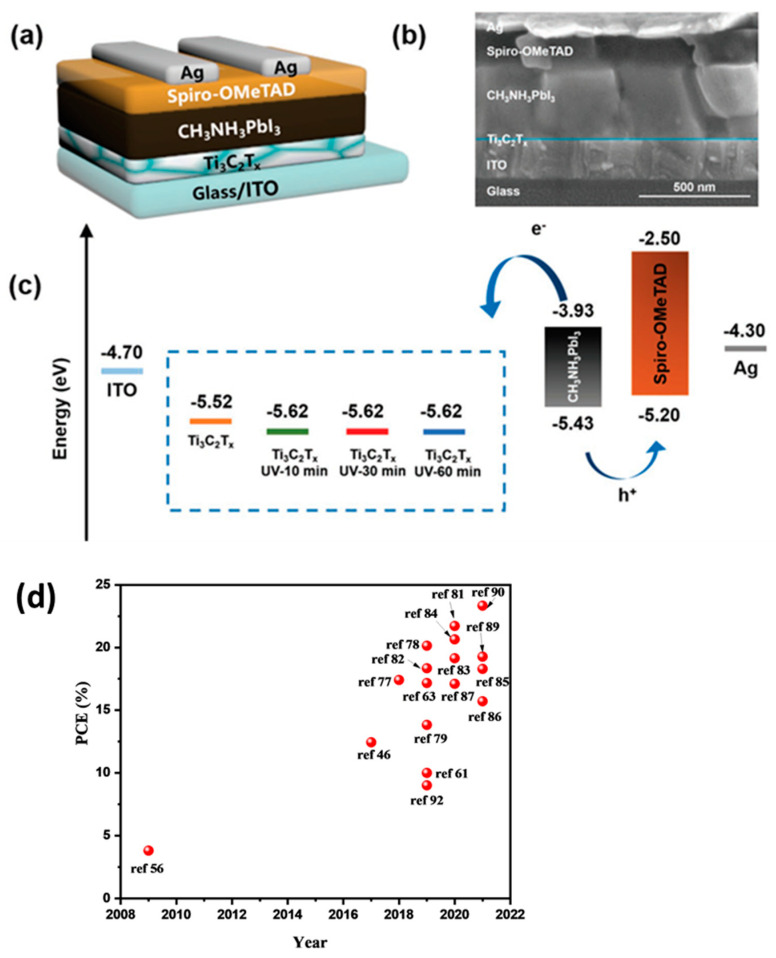
Schematic diagram (**a**), cross-sectional SEM (**b**) and energy level diagram (**c**) of the Ti_3_C_2_T*_x_* MXene-based PSCs. Reprinted with permissions from Ref. [63]. PCE (**d**) of previously published articles.

The developed PSCs device exhibited a high PCE of 20.6% with good stability up to 3 months. The introduction of Ti_3_C_2_T*_x_* MXene enhanced the device stability as well as photovoltaic performance. This suggested the potential application of Ti_3_C_2_T*_x_* MXene in the development of PSCs. In 2021, Yang et al. [85] prepared Ti_3_C_2_T*_x_* MXene and investigated its properties for photovoltaic applications. The schematic picture of the developed PSCs is presented in Figure 3d. The energy level values of the Ti_3_C_2_T*_x_* MXene and other components have been summarized in Figure 3d. Authors found that energy level values of the Ti_3_C_2_T*_x_* MXene are well-matched with the energy values of the ITO and TiO_2_. The developed PSCs using Ti_3_C_2_T*_x_* MXene showed the highest PCE of 18.29%. In other work, an interesting PCE of 15.71% was also obtained using Ti_3_C_2_T*_x_* MXene as dopant [86]. Yang et al. [63] developed the planar structured PSCs using Ti_3_C_2_T*_x_* MXene as an electron transport layer. The schematic diagram of the fabricated PSCs is depicted in Figure 4a. The cross-sectional SEM image of the developed PSCs is presented in Figure 4b, which is clearly showing the presence of interlayers. Yang et al. [63] investigated the effect of UV-ozone treatment. The observations showed that UV-ozone treatment slightly influenced the work function of the Ti_3_C_2_T*_x_* MXene (Figure 4c). The UV-ozone treatment enhanced the surface Ti-O bonds without affecting electron mobility, which suggested its potential use as an electron transport layer. The developed PSCs using Ti_3_C_2_T*_x_* MXene as an electron transport layer exhibited the excellent PCE of 17.17% [63]. In some other recent reports, Ge et al. [87] investigated the role of Ti_3_C_2_ quantum dots in PSCs and obtained the highest PCE of 16%.

### 3.3. Ti_3_C_2_T*_x_* MXene as HTL for PSCs

The high conductivity of Ti_3_C_2_T*_x_* MXene may also be useful for its potential applications as HTL for the development of PSCs. The high conductivity Ti_3_C_2_T*_x_* MXene may facilitate the hole extraction and transportation, which alternatively improve the efficiency of the PSCs. Thus, Ti_3_C_2_T*_x_* MXene can be used as HTL for the development of PSCs. Cao et al. [79] also developed electrodes using 2D MXenes for the construction of HTL-free PSCs applications. The MXene-incorporated electrode-based PSCs exhibited the good PCE of 13.8% [79]. Saranin et al. [88] developed inverted p-i-n PSCs using Ti_3_C_2_T*_x_* MXene decorated with NiO and obtained a PCE of 19.2%. The recent progress in the enhancement of the PCE is depicted in Figure 4d. The above results showed that Ti_3_C_2_T*_x_* MXene has the potential for photovoltaic applications. The photovoltaic performances of the previously reported PSCs with Ti_3_C_2_T*_x_* MXene are summarized in Table 1.

## 4. Electrochemical Sensing Applications

### 4.1. Electrochemical Properties of Ti_3_C_2_ MXene

The distinctive attributes of MXene (with Ti_3_C_2_ being the predominant variant) in contrast to other two-dimensional substances have led to its growing application in fabricating electrochemical sensors. These distinctive features can be outlined as follows.

MXene boasts a distinct benefit in terms of its high electrical conductivity, a crucial factor for enhancing the speed of electron transfer in heterogeneous reactions when compared to other 2D materials [94]. This exceptional conductivity characteristic forms a fundamental basis for its application in electrochemical sensors. The synthesis of MXene with favorable solution dispersibility and stability is straightforward. This is of paramount importance for creating electrochemical sensors, given that the predominant technique for preparing modified electrodes involves pre-preparation of a well-dispersed coating solution for drop-casting. MXene serves as a robust material for substrate applications in printing scenarios. The methods of printing and pre/post-patterned coating offer an array of uncomplicated, economically viable, adaptable, and environment-friendly manufacturing methods for devices. Printing facilitates intricate 3D structures and multi-functional qualities, highly sought after in diverse applications. Hence, the introduction of MXene could propel printing/coating towards a more potent tool for fabricating devices and advancing industrial processes [95]. Leveraging its high stretch capacity and compatibility with living organisms, MXene stands as an excellent substrate choice for producing flexible conductive platforms. These platforms hold significant potential in crafting wearable electrochemical sensors, a pressing need for health monitoring and clinical analysis. With its two-dimensional layered structure and distinctive surface featuring numerous chemical groups, MXene displays considerable promise for integration with various functional materials or biomolecules for diverse analytical objectives. Various MXene-based nanostructures showcase a range of distinct and vibrant properties, offering opportunities for designing electrochemical sensors or devices with assorted functions, particularly in the realm of novel ECL or PEC sensors [96]. MXene’s compatibility with biomolecules such as enzymes, proteins, and nucleic acids, coupled with its non-toxic nature, renders it as an excellent carrier in biosensors or biomedical applications. The exceptional photothermal conversion capability of MXene enables the realization of a dual-mode detection strategy in electrochemical sensor design. This expands the array of signal strategies available for electrochemical sensors, as evidenced by recent advancements in this field [97]. Herein, we have compiled recent works on the development of Ti_3_C_2_-based biosensors.

### 4.2. Ti_3_C_2_-Based Enzymatic Biosensors

**Figure 5:**
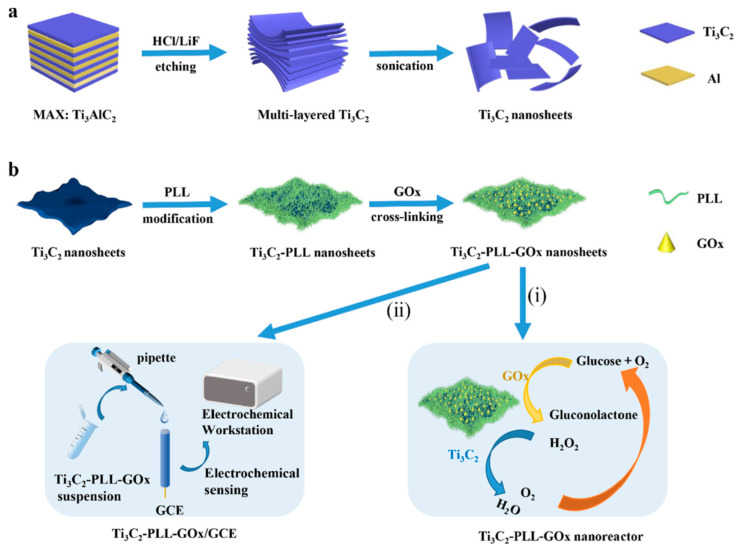
Schematic representation of the formation of the Ti_3_C_2_-PLL-GOx nanoreactor (**a**,**b**) and its application for cascade glucose oxidation (i) and electrochemical glucose sensing (ii) [57]. Reprinted with permission [57].

The fundamental aspect of constructing electrochemical biosensors involves the essential direct transfer of electrons (DET) between enzymes and electrodes. MXene materials exhibit a range of unique characteristics, such as a high specific surface area and remarkable electrical conductivity [98]. Hence, the integration of MXene could potentially serve as an effective approach to promote the electron transfer process. The pioneering MXene in this context, namely Ti_3_C_2_, was employed in developing electrochemical sensors. Notably, the inaugural Ti_3_C_2_-based electrochemical sensor, crafted in 2014, was an enzyme-based biosensor, designed to detect H_2_O_2_ [99]. In this regard, Ti_3_C_2_ MXene was utilized for immobilizing the enzyme hemoglobin (Hb). This immobilization showcased not only proficient enzyme immobilization capabilities but also furnished an advantageous microenvironment for sustaining the activity and stability of the protein. Furthermore, it facilitated the direct transfer of electrons within Hb, underscoring the efficacy of anchoring enzymes onto the surface of Ti_3_C_2_ MXene as a means to produce mediator-free enzyme-centric biosensors. Endeavors were also undertaken to immobilize other enzymes, such as acetylcholinesterase (AChE) and tyrosinase, onto the surface of Ti_3_C_2_ MXene [100,101]. These endeavors highlighted that Ti_3_C_2_ MXene, with its expansive specific surface area, favorable biocompatibility, hydrophilic surface, and exceptional metallic conductivity, indeed stands as a promising choice for serving as an excellent platform for immobilizing enzymes in the construction of enzyme-based biosensors. To enhance the performance of enzyme-based biosensors, many researchers have employed a strategy involving the integration of Ti_3_C_2_ MXene with various functional materials, particularly diverse nanomaterials, to form composite structures. For instance, Wang et al. improved the Ti_3_C_2_ MXene by attaching TiO_2_ nanoparticles (NPs), resulting in an increased surface area for protein adsorption and preservation of enzymatic stability and activity [102]. This modified Ti_3_C_2_ MXene was utilized to create a biosensor for Hb (hemoglobin), exhibiting superior detection capabilities for H_2_O_2_, with a limit of detection (LOD) of 14 nM, surpassing the performance of the TiO_2_NP-free biosensor. Moreover, Rakhi et al. developed a glucose oxidase (GOx)-based biosensor by constructing a nanocomposite of Au/Ti_3_C_2_ MXene [103]. The integration of Au NPs endowed the composite with distinctive electrocatalytic properties through synergistic effects. The resulting GOx/AuNPs/Ti_3_C_2_/Nafion/GCE biosensor showcased a relatively high amperometric sensitivity of 4.2 μA mM^−1^ cm^−2^ and an LOD of 5.9 μM for glucose detection. Similarly, Jiang et al. [104] employed an Ag@Ti_3_C_2_ nanocomposite as a carrier for AChE (acetylcholinesterase), fabricating a biosensor for malathion detection. In a separate study, Song et al. [105] developed an AChE-based biosensor for the identification of organophosphorus pesticide (OP) methamidophos. This biosensor was founded on a three-dimensional (3D) composite structure of MnO_2_@Mn_3_O_4_/MXene/AuNPs, achieving a remarkably low LOD of 0.134 pM for methamidophos. In the realm of glucose biosensing, the enzymatic process of glucose oxidation generates a potentially harmful byproduct, H_2_O_2_ [57]. This byproduct, however, frequently hinders the effectiveness of GOx (glucose oxidase) in practical applications. In order to address this concern, Wu, M. et al. devised a novel solution [57]. They engineered a hybrid nanoreactor utilizing a combination of Ti_3_C_2_, poly-L-lysine (PLL), and glucose oxidase (GOx) (Figure 5a,b). This nanoreactor exhibited the capability to drive both the sequential reactions of glucose oxidation and the subsequent breakdown of H_2_O_2_.

Interestingly, the Ti_3_C_2_ MXene component demonstrated proficiency in catalyzing the breakdown of H_2_O_2_. By incorporating GOx onto this platform, a cascading reaction for glucose oxidation was initiated. To realize this concept, the researchers fabricated Ti_3_C_2_/PLL/GOx nanoreactors, distinguished by their exceptional catalytic performance. These nanoreactors were then affixed to a glassy carbon electrode, creating a glucose biosensor with an impressive limit of detection (LOD) of 2.6 μM. Another notable advancement was accomplished by Wang et al. [106], who established a dual-enzyme biosensor for inosine monophosphate (IMP) detection. In this approach, Ti_3_C_2_ MXene was combined with Au@Pt nanoflowers to harness robust catalytic abilities. Subsequently, two enzymes (5′-nucleotidase and xanthine oxidase) were immobilized on this composite. The resultant biosensor displayed an LOD of 2.73 ng/mL for inosine monophosphate detection in meat samples. The Ti_3_C_2_ MXene has the potential to serve as a constituent in conductive foundations within electrode arrangements beyond the glassy carbon electrode (GCE). Researchers created a composite called Ti_3_C_2_/graphene oxide (Ti_3_C_2_-GO), which was employed in crafting an inkjet-printed biosensor for hydrogen peroxide detection. This study revealed that the printable Ti_3_C_2_-GO composite exhibited remarkable capabilities as a sensing platform for electrochemical analysis [107]. Additionally, they developed a hybrid composite, Pt/PANI/MXene, by combining platinum particles, polyaniline, and Ti_3_C_2_ MXene [108]. This composite was utilized to modify a screen-printed carbon electrode (SPCE) to formulate a biosensor capable of detecting both hydrogen peroxide and lactate. The resulting SPCE demonstrated a low detection limit of 1.0 μM for H_2_O_2_. Following the immobilization of lactate oxidase, the biosensor facilitated lactate detection through amperometric measurements, achieving a detection limit of 5.0 μM for lactate. This biosensor proved its applicability for lactate measurement in milk samples, showcasing strong durability and dependability. The reported biosensors using Ti_3_C_2_ are compiled in Table 2.

**Figure 6:**
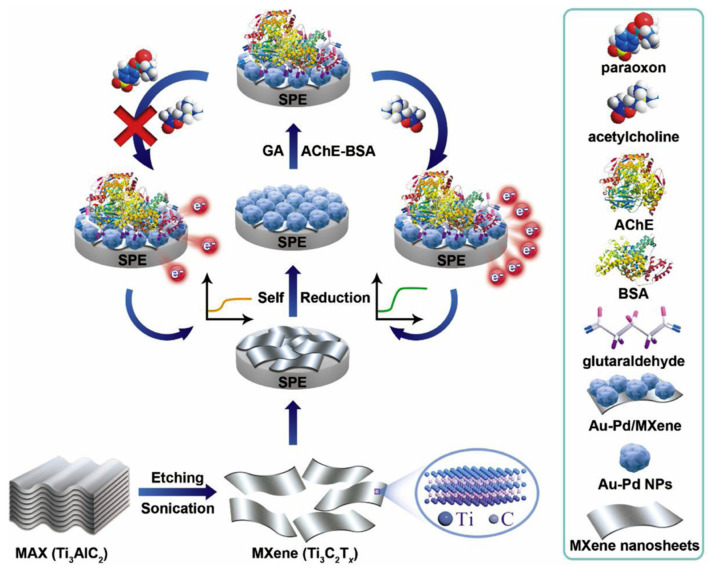
Schematic representation for the preparation of Ti_3_C_2_T*_x_* nanosheets and fabrication of enzyme-based pesticide biosensor (Ti_3_C_2_T*_x_*/Au-Pd). Reprinted with permission [60].

Zhao et al. [60] also fabricated a biosensor for the determination of pesticides. Figure 6 exhibits the fabrication of the biosensor. In this research, bimetallic nanoparticles consisting of a combination of gold and palladium (Au-Pd NPs) were synthesized through self-reduction occurring on the surface of ultrathin MXene nanosheets (Ti_3_C_2_T*_x_*).

The resulting multi-dimensional nanocomposites (MXene/Au-Pd) demonstrate excellent conductivity and stability that prove advantageous for facilitating electron transfer and enzyme immobilization. By incorporating these nanocomposites into a disposable screen-printed electrode (SPE), a high-performance enzymatic biosensor was developed for swiftly detecting organophosphates (OPs). The electrochemical platform relies on the use of MXene/Au-Pd nanocomposites, as illustrated in Figure 6. The specific model pesticide chosen for this study was paraoxon, due to its high toxicity and the potential for its conversion from other OPs.

**Figure 7:**
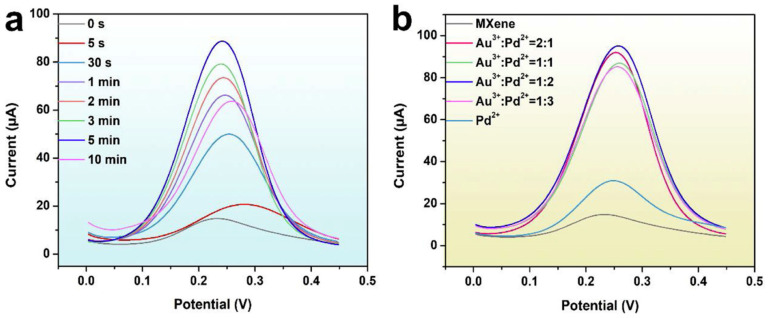
DPV response of SPE/MXene/Au-Pd with (**a**) different growth time of Au-Pd NPs and (**b**) different concentration ratios of Au^3+^ to Pd^2+^ in 0.1 M KCl solution containing 5.0 mM K_3_[Fe(CN)_6_] [60]. Reprinted with permission [60].

Figure 7a,b depict the Differential Pulse Voltammetry (DPV) outcomes of the SPE/MXene/Au-Pd under varying conditions: (a) diverse durations of Au-Pd NP growth, and (b) distinct ratios of Au^3+^ to Pd^2+^ concentrations, all conducted in a 0.1 M KCl solution containing 5.0 mM K_3_[Fe(CN)_6_]. In Figure 7a, the electric current response progressively rises until the 5-min mark, beyond which it declines. This decrease is possibly attributable to the congestion of NPs, which affects the kinetics of electron transfer. Evaluating both the morphology of the NPs and the DPV response, the researchers selected a growth period of 5 min as the optimal timeframe for Au-Pd NP formation. Additionally, the researchers fine-tuned the ratio of Au^3+^ to Pd^2+^ precursor concentrations. The most substantial DPV response occurs when the ratio stands at 1:2, as demonstrated in Figure 7b. Considering these findings, the researchers opted for the 1:2 concentration ratio for the Au^3+^-Pd^2+^ precursor. The resultant sensor displayed commendable performance.

### 4.3. Electrochemical Non-Biosensors

**Figure 8:**
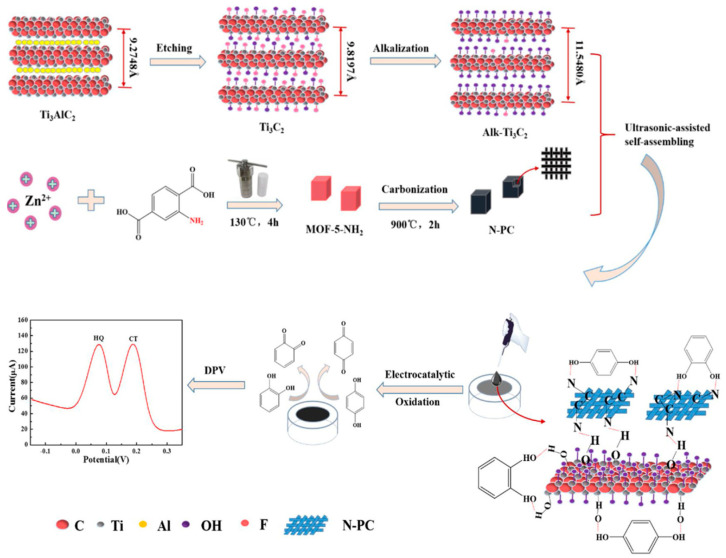
Schematic description of the preparation and application of alk-Ti_3_C_2_/N-PC/GCE [123]. Reprinted with permission [123].

MXene could be utilized in creating electrochemical non-biosensors which do not require the immobilization of enzymes. In 2018, the initial electrochemical non-biosensor utilizing pristine Ti_3_C_2_ MXene was manufactured. This sensor was designed to detect the contaminant BrO_3_^−^ in drinking water [109]. In this application, Ti_3_C_2_ MXene served as both a signal-enhancing matrix and a reducing agent, displaying remarkable electrocatalytic characteristics that facilitated effective reduction of BrO_3_^−^. Following this, two electrochemical sensors similar to the first one were developed using Ti_3_C_2_ MXene-modified glassy carbon electrodes (GCE). These sensors were used for the detection of the pesticide carbendazim [110] and the neurotransmitter dopamine [111]. In the case of carbendazim detection, the sensor achieved carbendazim redox at lower overpotentials compared to a graphene-based sensor [112]. The dopamine sensor demonstrated robust sensitivity, with a limit of detection (LOD) of around 3 nM for dopamine detection in actual samples. The incorporation of MXenes into other electrode systems, such as graphite composite paste electrodes (GCPE) [113] and screen-printed electrodes (SPE) [114], was also explored. An MXene/GCPE electrochemical sensor was devised for the detection of adrenaline. Notably, this was the first instance of introducing Ti_2_C MXene into an electrochemical sensing system. The developed sensor achieved an LOD of 9.5 nM and could be further applied for detecting adrenaline in pharmaceutical samples, recovering between 99.2 and 100.8%. Another MXene-based electrochemical sensor was created using a screen-printed electrode (SPE) configuration for simultaneous voltammetric determination of acetaminophen (ACOP) and isoniazid (INZ). The Ti_3_C_2_ MXene displayed excellent electrocatalytic performance in the oxidation of ACOP and INZ compared to a bare SPE electrode in 0.1 M H_2_SO_4_. The distinct oxidation peak potentials allowed simultaneous detection of both targets. Consequently, this sensor attained LODs of 0.048 μM and 0.064 mM for ACOP and INZ, respectively. Additionally, the utilization of Ti_3_C_2_ MXene extended to the creation of various composites or combinations involving alternative substances or molecules, serving distinct analytical objectives. For instance, a hybrid of NiO and Ti_3_C_2_ was employed for detecting H_2_O_2_ without enzyme reliance [115]. Another example involves a three-dimensional porous composite of Ti_3_C_2_ and NiCo-LDH (Nickel-Cobalt layered double hydroxide) for glucose sensing without enzymes [116]. Furthermore, a composite of Au NPs and Ti_3_C_2_ was devised to sensitively detect nitrite [117]. A nanocomposite featuring Pd deposited on Ti_3_C_2_ was developed for swift, real-time detection of l-cysteine (l-Cys) [118]. A composite of Mn_3_(PO_4_)_2_ and Ti_3_C_2_, synthesized using adenosine triphosphate (ATP) as a template, was applied for amperometric detection of superoxide anions O_2_^•−^ released from HepG2 cells [119]. Another innovation involved a self-assembled nanocomposite of Ti_3_C_2_ and MWCNTs (multi-walled carbon nanotubes) for simultaneous electrochemical detection of hydroquinone (HQ) and catechol (CT) [120]. Moreover, a composite of methylene blue (MB), Cu NPs, and Ti_3_C_2_ was created for ratiometric electrochemical detection of piroxicam [121]. Throughout these studies, the performance of Ti_3_C_2_ MXene consistently proved highly effective in the design and operation of electrochemical sensors. The tendency of MXene to stack together is driven by hydrogen bonding and van der Waals interactions among its layers. This stacking can significantly reduce the effective surface area, thereby limiting its electrochemical performance. To counteract this issue, researchers like Tu et al. [122] introduced carbon nanohorns (CNHs) as spacers and created Ti_3_C_2_/CNHs nanocomposites. This layered MXene/CNHs structure displayed excellent conductivity, enhanced catalytic activity, and increased pathways for ion diffusion. Using this nanocomposite, they developed an electrochemical sensor for carbendazim with an impressive low detection limit of 1.0 nM. Similarly, Huang et al. [123] employed nitrogen-doped porous carbon derived from MOF-5-NH_2_ (N-PC) as a spacer to prevent restacking of Ti_3_C_2_ MXene sheets, as shown in Figure 8.

**Figure 9:**
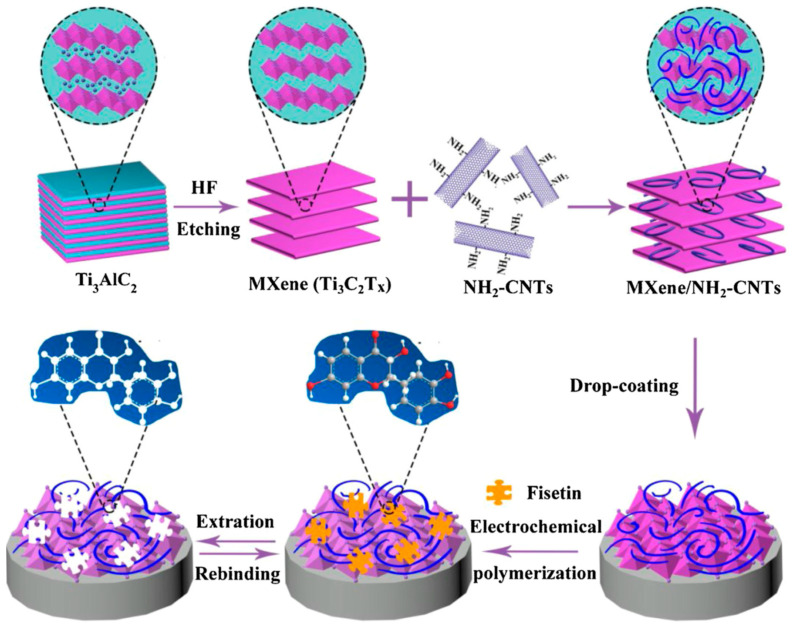
Schematic illustration for the preparation of MIP/MXene/NH_2_-CNTs/GCE and the adsorption mechanism in the imprinted cavity [124]. Reprinted with permission [124].

Authors fabricated an alk-Ti_3_C_2_/N-PC electrochemical sensor for detecting hydroquinone (HQ) and catechol (CT) in industrial wastewater. In addition to using N-PC to prevent restacking, they treated the Ti_3_C_2_ MXene sheets with an alkaline intercalation process. This treatment led to the presence of abundant -OH groups on the MXene surface, which facilitated hydrogen-bond interactions for sensing HQ and CT. The resulting alk-Ti_3_C_2_/N-PC electrochemical sensor displayed low detection limits of 4.8 nM for HQ and 3.1 nM for CT, covering a broad linear range from 0.5 to 150 μM. Selectivity poses a challenge in the practical use of electrochemical sensors for targeted measurements. Molecularly imprinted polymers (MIPs) offer a solution to enhance sensor selectivity through specific recognition properties, cost-effectiveness, and quick synthesis. Ma et al. [124] developed a sensitive and selective electrochemical sensor for detecting fisetin using a hierarchical porous Ti_3_C_2_ MXene/amino carbon nanotubes (MXene/NH_2_-CNTs) composite combined with MIP (Figure 9).

The composite was formed by assembling negatively charged Ti_3_C_2_ flakes and positively charged NH_2_-CNTs. The amino-functionalized CNTs not only provided good conductivity but also introduced positive charges on their surface, acting as spacers to prevent Ti_3_C_2_ MXene aggregation. The resultant MIP/Ti_3_C_2_ MXene/NH_2_-CNTs/GCE sensor exhibited excellent analytical performance for fisetin detection, achieving a low LOD of 1.0 nM. Additionally, numerous advancements have been made in the field of electrochemical non-biosensors utilizing MXene-based materials. To facilitate the real-time and in situ detection of hydrogen peroxide (H_2_O_2_) released by living cells, Dang and colleagues [125] developed an electrochemical biosensor. This biosensor was constructed using titanium carbide (Ti_3_C_2_) nanosheets intercalated with Prussian blue nanoparticles (PB NPs/Ti_3_C_2_), achieving a notably low limit of detection (0.20 μM) for H_2_O_2_. Moreover, the PB NPs/Ti_3_C_2_ composite exhibited minimal harm to normal fibroblast cells across different time intervals and concentrations, underscoring its potential applicability in areas concerning living cells. In the realm of sulfadiazine detection, Kokulnathan et al. [126] synthesized nanocomposites of Ti_3_C_2_ and boron nitride (Ti_3_C_2_/BN) and designed an electrochemical catalytic sensor by modifying an electrode with Ti_3_C_2_/BN. This sensor exhibited a remarkably sensitive limit of detection (3.0 nM) for sulfadiazine. Kalambate and co-workers [127] developed an electrochemical sensor capable of detecting ifosfamide (IFO), acetaminophen (ACOP), domperidone (DOM), and sumatriptan (SUM). This sensor relied on a self-assembled nanocomposite thin film consisting of MXene, multi-walled carbon nanotubes (MWCNT), and chitosan. The limits of detection achieved for IFO, ACOP, DOM, and SUM were 0.00031, 0.00028, 0.00034, and 0.00042 μM, respectively. The reported sensors are summarized in Table 3.

## 5. Conclusions and Future Perspective

MXenes, especially Ti_3_C_2_T*_x_* MXene, possess unique optoelectronic properties, and an adjustable composition/structure. The Ti_3_C_2_T*_x_* MXene is one of the most important 2D materials which has been used in various applications. The work function of the Ti_3_C_2_T*_x_* MXene can be easily tuned, which makes it the most suitable candidate as an electron and hole transport layer for perovskite solar cells. In the last 3–4 years, Ti_3_C_2_T*_x_* MXene has been used as an additive, electron/hole transport layers, and electrode materials for the development of perovskite solar cells. The reported literature showed that the incorporation of Ti_3_C_2_T*_x_* MXene not only enhances the photovoltaic performance of the perovskite solar cells but also improves long term stability. Therefore, it is clear that it would be beneficial to develop the novel device architectures of the perovskite solar cells with Ti_3_C_2_T*_x_* MXene-assisted metal oxide as an electron transport layer. This may improve the photovoltaic performance as well as long term stability of the perovskite solar cells. In the realm of future applications of MXene in electrochemical sensors, there are several potential research avenues. Firstly, in the context of biosensors, MXene is often utilized as a carrier for biomolecules. The common methods for immobilizing biomolecules on MXene surfaces involve the assistance of Au nanoparticles or electrostatic adsorption. However, we believe that establishing a direct chemical linkage between biomolecules and MXene holds greater value due to the enhanced stability of resulting biocomposites and the rich surface chemistry of MXene. This underscores the significance of surface functionalization techniques like amination or carboxylation of MXene. Further exploration of diverse combinations in this regard is warranted. In summary, leveraging MXene as a powerful tool opens up the possibility of developing more advanced electrochemical sensors in the future.

## Figures and Tables

**Table 1:** Photovoltaic performance of the recently reported PSCs.

S. No.	Device Structure	Voc (V)	Jsc (mA/cm^2^)	FF	PCE (%)	References
1.	ITO/Ti_3_C_2_T*_x_*/CH_3_NH_3_PbI_3_/ Spiro-OMeTAD/Ag	1.08	22.63	0.70	17.17	[63]
2.	ITO/SnO_2_/perovskite: Ti_3_C_2_T*_x_*/Spiro-MeOTAD/Au	1.03	22.26	0.76	17.41	[77]
3.	FTO/c-TiO_2_ +MXene/ m-TiO_2_ + MXene/MXene/perovskite + MXene/ spiro-OMeTAD/Au	1.09	23.82	0.77	20.14	[78]
4.	FTO/TiO_2_/CH_3_NH_3_PbI_3_/ MXene	0.95	22.96	0.63	13.83	[79]
5.	c-TiO_2_/m-TiO_2_-TQD/perovskite/ Spiro-OMeTAD-Cu_1.8_S	1.13	23.64	0.77	21.72	[81]
6	ITO/SnO_2_-Ti_3_C_2_ MXene/MAPbI_3_/Spiro-OMeTAD/ Ag	1.06	23.14	0.75	18.34	[82]
7.	FTO/SnO_2_-MXene/ (FAPbI_3_)_0.97_(MAPbBr_3_)_0.03_/spiro-OMeTAD	1.07	24.52	0.77	19.14	[83]
8.	FTO/MXene-SnO_2_/Perovskite/Spiro- OMeTAD/Au	1.11	24.34	-	20.65	[84]
9.	ITO/HO-Ti_3_C_2_T*_x_*@Ti_3_C_2_T*_x_*/CH_3_NH_3_PbI_3_/ Spiro-OMeTAD/Ag	1.07	23.11	0.74	18.29	[85]
10.	ITO/SnO_2_/(BA)_2_(MA)_4_Pb_5_I_16_-Ti_3_C_2_ MXene/Spiro-OMeTAD/Ag	1.11	20.87	0.67	15.71	[86]
11.	FTO/c-TiO_2_/ m-TiO_2_-2D MXene/ perovskite-0D Ti_3_C_2_ QDs/Spiro-OMeTAD/Au	0.92	19.6	0.66	17.1	[87]
12.	FTO/SnO_2_/perovskite: Ti_3_C_2_T*_x_*/Spiro-MeOTAD/Au	1.12	23.48	0.73	19.27	[89]
13.	ITO/SnO_2_-MQDs/perovskite/ Spiro/MoO_3_/Au	1.17	24.96	0.79	23.34	[90]
14.	FTO/Ti_3_C_2_T*_x_*@TiO_2_ (0.2 wt%)/Cs_2_AgBiBr_6_/Spiro/MoO_3_/Ag	0.96	4.14	0.70	2.81	[91]
15.	FTO/TiO_2_/CsPbBr_3_/ Ti_3_C_2_-MXene/C	1.44	8.54	0.73	9.01	[92]
16.	FTO/c-TiO_2_/CsPbBr_3_/C+ CNTs+MXene	1.35	7.16	0.72	7.09	[93]

**Table 2:** Sensing performance for enzymatic biosensors.

Materials	Sensing Analyte	Sensing Technique	Linear Range	LOD	References
Ti_3_C_2_	Glucose	CV	0.02–1.1; 4.0–20 mM	2.6 μM	[57]
Ti_3_C_2_	β-hydroxybutyrate	Amperometric	0.36–17.9 mM	45 μM	[58]
Ti_3_C_2_	Glucose	Amperometric	50–27,750 μM	23 μM	[59]
Ti_3_C_2_/Au-PdNPs	Paraoxon	Amperometric	0.1–1000 μg/L	1.75 ng/L	[60]

**Table 3:** Sensing performance for non-biosensors.

Materials	Sensing Analyte	Sensing Technique	Linear Range	LOD	References
Ti_3_C_2_/PBNPs	Hydroquinone (HQ)	DPV	-	4.8 nM	[123]
Ti_3_C_2_/PBNPs	Catechol (CT)	DPV	0.5–150 μM	3.1 nM	[123]
Ti_3_C_2_/NH_2_-CNTs	Fisetin	DPV	0.003–20.0 μM	1 nM	[124]
Ti_3_C_2_/BN	Sulfadiazine	DPV	0.01–44; 59–186 μM	3 nM	[126]
Ti_3_C_2_/MWCNT	Ifosfamide (IFO)	Adsorptive Stripping	0.0011–1.0 μM	0.00031 μM	[127]
Ti_3_C_2_/AuNPs	Folic acid (FA)	Amperometric	0.02–3580 μM	6.2 nM	[128]
Ti_3_C_2_/AuNPs	Uric acid (UA)	Amperometric	0.03–1520 μM	11.5 nM	[128]
Ti_3_C_2_/TiO_2_	NO_2_^−^	DPV	0.003–0.25 mM	850 nM	[129]
Ti_3_C_2_/BiNPs	Pb^2+^	SWV	0.06–0.6 μM	10.8 nM	[130]
Ti_3_C_2_/ZIF-8	Hydrazine	Amperometric	10 μm to 7.7 mM	5100	[131]

## Data Availability

No new data were created, or where data is unavailable due to privacy or ethical restrictions.
